# Numerical Investigation of Odor-Guided Navigation in Flying Insects: Impact of Turbulence, Wingbeat-Induced Flow, and Schmidt Number on Odor Plume Structures

**DOI:** 10.3390/biomimetics8080593

**Published:** 2023-12-06

**Authors:** Menglong Lei, Mark A. Willis, Bryan E. Schmidt, Chengyu Li

**Affiliations:** 1 Department of Mechanical Engineering, Villanova University, Villanova, PA 19085, USA; mlei@villanova.edu; 2 Department of Biology, Case Western Reserve University, Cleveland, OH 44106, USA; mark.willis@case.edu; 3 Department of Mechanical and Aerospace Engineering, Case Western Reserve University, Cleveland, OH 44106, USA; bryan.e.schmidt@case.edu

**Keywords:** flapping wings, unsteady aerodynamics, turbulence, wingbeat-induced airflow, olfaction in insects

## Abstract

Odor-guided navigation is fundamental to the survival and reproductive success of many flying insects. Despite its biological importance, the mechanics of how insects sense and interpret odor plumes in the presence of complex flow fields remain poorly understood. This study employs numerical simulations to investigate the influence of turbulence, wingbeat-induced flow, and Schmidt number on the structure and perception of odor plumes by flying insects. Using an in-house computational fluid dynamics solver based on the immersed-boundary method, we solve the three-dimensional Navier–Stokes equations to model the flow field. The solver is coupled with the equations of motion for passive flapping wings to emulate wingbeat-induced flow. The odor landscape is then determined by solving the odor advection–diffusion equation. By employing a synthetic isotropic turbulence generator, we introduce turbulence into the flow field to examine its impact on odor plume structures. Our findings reveal that both turbulence and wingbeat-induced flow substantially affect odor plume characteristics. Turbulence introduces fluctuations and perturbations in the plume, while wingbeat-induced flow draws the odorant closer to the insect’s antennae. Moreover, we demonstrate that the Schmidt number, which affects odorant diffusivity, plays a significant role in odor detectability. Specifically, at high Schmidt numbers, larger fluctuations in odor sensitivity are observed, which may be exploited by insects to differentiate between various odorant volatiles emanating from the same source. This study provides new insights into the complex interplay between fluid dynamics and sensory biology and behavior, enhancing our understanding of how flying insects successfully navigate using olfactory cues in turbulent environments.

## 1. Introduction

The ability of flying insects to navigate complex environments using olfactory cues is a remarkable feat that has captured the attention of researchers in various disciplines, ranging from biology to engineering [1,2,3,4]. Odor-guided navigation is crucial for a variety of essential activities, such as foraging for food, locating mates, and avoiding predators [5]. Understanding the mechanisms underlying this odor-guided navigation can not only shed light on intricate biological processes but also inspire engineering solutions for artificial sensing and navigation systems.

One of the key challenges in this research domain lies in the inherent complexity of fluid flow conditions that insects encounter. In nature, turbulent air flows can significantly distort and fragment odor plumes, transforming them into complex, fluctuating filaments [6,7]. These turbulent conditions introduce variability in the plume structure, rendering the olfactory-guided navigation of insects a challenging task [8]. Although efforts have been made to visualize odor plumes via techniques like photoionization detectors (PID) [9], fluorescein dye [10], and planar laser-induced fluorescence (PLIF) [11,12], these studies have struggled to simultaneously capture both the fluid dynamics and detailed odor plume structures at the microscale level relevant to insects. Early research led by Wright [13] used Gaussian plume models to estimate average pheromone concentrations downwind of an odor source. As one might expect, the average concentration decreases with the downwind distance from the source, and the average concentration on the midline of the plume decays with a power law. However, later studies [14,15] indicated that insects do not respond to the mean concentration as predicted by the Gaussian distribution model. Instead, insects respond to instantaneous concentrations that are frequently many-fold higher than the mean concentrations. More recent experimental studies have focused on capturing these instantaneous concentrations and their dispersal patterns, revealing a probability density function model that can describe the fluctuating odor landscape adequately, including statistics of the fluctuations [11,12,16,17]. In addition to the fluctuations, the discontinuous nature of odor filaments causes intermittency in the concentration. Celani et al. [6] used a Lagrangian approach to solve the advection–diffusion equations and demonstrated that intermittency is a function of downwind distance from the source. Their results illustrate that the distributions of whiff durations and blank durations follow a power law with an exponent of −3/2.

Despite considerable experimental and theoretical studies on the spatiotemporal structures of odor landscapes; however, no attempts have been made to incorporate the effects of wingbeat-induced flow into a predictive model for concentration fluctuations and intermittency. A number of experimental [18,19] and numerical studies [3,20] indicate that the local odor concentration can be significantly enhanced by the induced flow generated by flapping wings [21]. One might expect that the unsteady flow generated by flapping wings would perturb the odor plume structure and odorant transport around the olfactory receptors of insects (i.e., antennae), and thus impact how the insects perceive the global odor landscape. To date, it still remains unclear how wingbeat-induced flow affects the spatiotemporal structure of the odor landscape used to track wind borne odors by flying insects.

Insects demonstrate a remarkable sensitivity to odors, generating odorant-evoked spiking responses in their olfactory neurons within 3 ms [22], and achieving a temporal resolution as high as 100 Hz through their antennae [23]. This high-resolution olfactory sensing enables insects to pick up subtle fluctuations in odor plumes and modify their navigational strategies accordingly. For example, fruit flies have the ability to modify their behavior based on prior experiences, learning from their environment and adapting to better navigate challenges and opportunities [24], while mosquitoes [25] and moths [26] are observed to engage in more upwind flights when exposed to fluctuating plumes. Furthermore, the Schmidt number, a dimensionless parameter representing the ratio of momentum diffusivity to mass diffusivity, emerges as another key factor in understanding odorant transport phenomenon in insects’ flights. While existing research has highlighted the importance of the Schmidt number of an odorant in affecting olfactory sensitivity in aquatic organisms [27], its role in the airborne olfaction of flying insects remains largely unexplored. Numerical simulations offer a promising avenue for such investigations, yet they have been underrepresented in the literature to date. Previous studies have investigated the impact of turbulence on insects’ forward flight, using pre-generated velocity fields to model turbulent conditions [28,29]. These studies concluded that turbulence introduces variabilities in aerodynamic forces but does not significantly affect the overall, time-averaged flight performance. However, the potential interplay between these flow fluctuations and odor plume structures, and consequently, their influence on olfactory perception, has yet to be investigated.

In the present study, we leverage advanced numerical simulations to conduct an in-depth analysis of the factors affecting odor plume structures in flying insects, specifically focusing on the roles of turbulence, wingbeat-induced flow, and the Schmidt number. Utilizing an in-house, three-dimensional computational fluid dynamics (CFD) solver based on the immersed boundary method, we first solved the Navier–Stokes equations in conjunction with the equations of motion for passive pitching wings, thereby obtaining the flow field. Subsequently, the odor advection–diffusion equation is solved at each time step to compute the odor concentration field. We employ a fruit fly model as our insect of interest, primarily due to the extensive experimental data available concerning its odor-tracking flight and its relatively straightforward wing morphology, which eliminates complicating factors associated with wing deformation. Realistic wing kinematics are generated by incorporating aerodynamic, elastic, inertial, and gravitational forces into a torsional spring model that calculates the wing’s pitch angle. Within a simulated wind tunnel environment, the fruit fly model is positioned in a constant air flow, upstream of an odor source emitting a consistent concentration of odorants. To simulate turbulence, a pre-calculated turbulence field is imposed at the point where the odor plumes are released. Through this comprehensive approach, our work aims to provide new insights into the interplay between fluid dynamics and sensory biology and behavior, enriching our understanding of how flying insects successfully employ olfactory cues for navigation in turbulent environments.

## 2. Methodology

### 2.1. Governing Equations and Numerical Method

The three-dimensional viscous incompressible Navier–Stokes equations are solved using an in-house immersed-boundary-method-based CFD solver. The equations are written in tensor form:

(1) $$\{\begin{array}{c} \frac{\partial u_{i}}{\partial x_{i}}=0\\ \frac{\partial u_{i}}{\partial t}+\frac{\partial \left(u_{i}u_{j}\right)}{\partial x_{j}}=-\frac{1}{\rho }\frac{\partial P}{\partial x_{i}}+\nu \frac{\partial }{\partial x_{j}}\left(\frac{\partial u_{i}}{\partial x_{j}}\right) \end{array}$$

where *u_i_* are the velocity components, *P* is the pressure, *ρ* is the fluid density, and *ν* is the kinematic viscosity.

**Figure 1:**
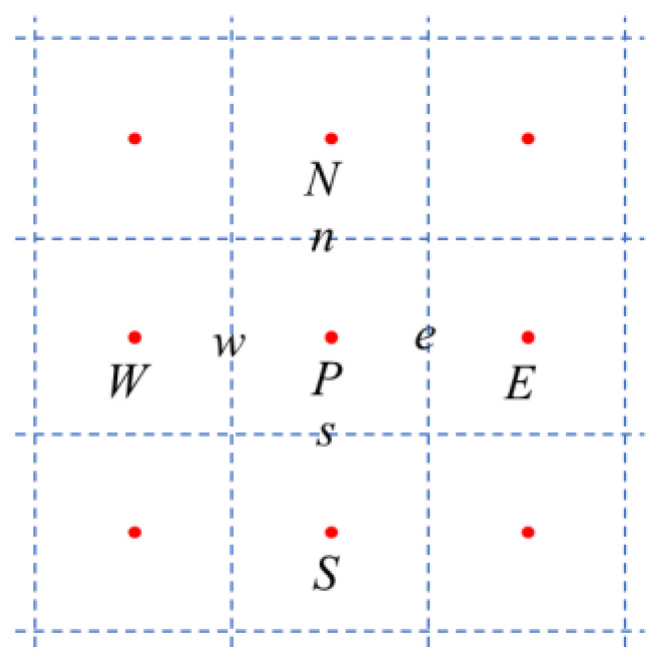
Schematic of the grid. The uppercase letters denote cell-centered variables, the lowercase letters denote the face-centered variables.

The above equations are discretized using a cell-centered, collocated arrangement of the primitive variables, and are solved using a finite-difference-based immersed-boundary method [30] in a non-body-conforming Cartesian grid, as shown in Figure 1. The uppercase letters, *W*, *E*, *N*, and *S*, represent cell-centered variables, and the lowercase letters, *w*, *e*, *n*, and *s*, represent face-centered variables that are calculated by interpolating the corresponding cell-centered variables. A second-order central difference scheme is employed in space to discretize the advection and diffusion terms. Boundary conditions on the immersed boundaries are imposed through a ghost-cell procedure. The equations are integrated with time using the fractional step method. The immersed-boundary-method eliminates complex remeshing algorithms for moving boundaries on body-conforming grids at each time step. Our CFD solver has been successfully applied to study canonical revolving wings flapping propulsion problems and insect flight [3]. Details of the CFD solver in solving Navier–Stokes equations were elaborated and validated in our previous studies [31].

### 2.2. Odor Advection–Diffusion Equation

At each time step, based on the velocity field, the odor advection–diffusion equation is solved to obtain the odor concentration landscape. The odor advection–diffusion equation is discretized using an implicit method (Equation (3)) with second-order accuracy in space.

(2) $$\frac{\partial C}{\partial t}+u_{i}\frac{\partial C}{\partial x_{i}}=D\frac{\partial^{2}C}{\partial x_{i}^{2}}$$

(3) $$\frac{C^{n+1}-C^{n}}{\Delta t}+\frac{\delta C^{n}U_{i}^{n}}{\delta x_{i}}=D\frac{\delta }{\delta x_{i}}\left(\frac{\delta C^{n+1}}{\delta x_{i}}\right)$$

where *C* is the odor intensity, *D* is the odor diffusivity, and *U_i_* is the face-centered velocity obtained from interpolation of the cell-centered velocity *u_i_*.

The odor advection–diffusion equation is discretized on the same non-body-conforming Cartesian grid as the Navier–Stokes equations solver, as shown in Figure 1. The differential equation for odor transportation can be written as

(4) $$\begin{array}{c} C_{P}^{n+1}-D\Delta t\left(a_{W}C_{W}^{n+1}+a_{E}C_{E}^{n+1}+a_{N}C_{N}^{n+1}+a_{S}C_{S}^{n+1}+a_{B}C_{B}^{n+1}+a_{F}C_{F}^{n+1}+a_{P}C_{P}^{n+1}\right)\\ =C_{P}^{n}-\Delta t\left(\frac{C_{e}^{n}U_{e}^{n}-C_{w}^{n}U_{w}^{n}}{\Delta x}+\frac{C_{n}^{n}U_{n}^{n}-C_{s}^{n}U_{s}^{n}}{\Delta y}+\frac{C_{f}^{n}U_{f}^{n}-C_{b}^{n}U_{b}^{n}}{\Delta z}\right) \end{array}$$

where the coefficients, *a_W_*, *a_E_*, *a_N_*, *a_S_*, *a_B_*, *a_F_*, *a_P_*, are calculated by discretizing the following diffusion term:

(5) $$\begin{array}{c} \frac{\delta }{\delta x_{i}}\left(\frac{\delta C}{\delta x_{i}}\right)=\frac{\frac{C_{E}-C_{P}}{\Delta x}-\frac{C_{P}-C_{W}}{\Delta x}}{\Delta x}+\frac{\frac{C_{N}-C_{P}}{\Delta y}-\frac{C_{P}-C_{S}}{\Delta y}}{\Delta y}+\frac{\frac{C_{F}-C_{P}}{\Delta z}-\frac{C_{P}-C_{B}}{\Delta z}}{\Delta z}\\ =a_{E}C_{E}+a_{W}C_{W}+a_{N}C_{N}+a_{S}C_{S}+a_{F}C_{F}+a_{B}C_{B}+a_{P}C_{P} \end{array}$$

This approach has been applied to investigate the odor plume structure perturbed by the canonical pitching–plunging motion and flapping wings of insects.

### 2.3. Wing Model with Torsional Spring

It has been demonstrated that insect wings can achieve the correct fundamental kinematic motion by passively responding to aerodynamic and wing inertia forces [32,33]. In the current study, the wing pitch angle is calculated using a torsional spring model integrated with the Navier–Stokes CFD solver using a two-way coupling method. As the wing flaps back and forth in the stroke plane, with a prescribed stroke angle and the wing root acting like a torsional spring, the wing pitches passively about the wing’s leading edge. This passive pitching model has been validated with real fruit flies and is used to provide a reasonable wing kinematics under various simulation setups.

**Figure 2:**
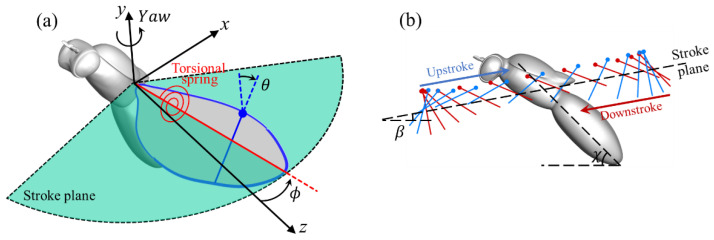
Schematic of the flapping wing with a torsional spring (**a**) and wing chord diagram during upstroke and downstroke (**b**), where *θ* is the pitch angle, *ϕ* is the stroke angle, *β* = 10° is the stroke plane angle, and *χ* = 45° is the body incline angle.

The fruit fly model adopted here has been used in our previous studies [3]. The aspect ratio of the wing is 3.2, defined as (span)^2^/(area). As shown in Figure 2a, the kinematics of the wing are defined by stroke angle (ϕ) and pitch (*θ*) angle with zero deviation in the angle with respect to the wing stroke plane. In Figure 2b, the stroke plane angle *β* is 10° and the inclination angle of the insect body *χ* is 45°. This stroke plane angle is specifically chosen by performing a series of test cases so that the drag generated during the downstroke approximately balances with the thrust generated during the upstroke for the baseline case.

The wing stroke angle is prescribed using Equation (6), while the wing deviation angle is fixed as zero during the entire flapping motion.

(6) $$\phi (t)=-A_{\phi }\cos(2\pi f_{}t)$$

where *ϕ*(*t*) is the instantaneous wing stroke angle at time *t*, *A_ϕ_*= 140° is the stroke amplitude, and *f* is the flapping frequency.

At each time step, the instantaneous inertia, aerodynamic, elastic, and gravity momentum are calculated, and the passive pitch angular acceleration of the wings about the wing leading edge is then obtained using the equation of motion, which is written as follows:

(7) $$\begin{array}{c} I_{xx}{\dot{\omega }}_{x}+\left(I_{zz}-I_{yy}\right)\omega_{y}\omega_{z}+I_{xy}\left(\omega_{x}\omega_{z}-{\dot{\omega }}_{y}\right)+I_{yz}\left(\omega_{z}^{2}-\omega_{y}^{2}\right)-I_{xz}\left({\dot{\omega }}_{z}+\omega_{x}\omega_{y}\right)\\ =M_{aero}+M_{elastic}+M_{gravity} \end{array}$$

where *I_xx_*, *I_yy_*, *I_zz_*, *I_xy_*, *I_xx_*, and *I_xz_* are the momentum of inertia of the wing. *M_aero_*, *M_elastic_*, and *M_gravity_* are the momentum due to aerodynamic, elastic, and gravitational forces, respectively. The aerodynamic forces are obtained by integrating the pressure and shear on the surface of the insect.

The nondimensional torsional stiffness of the wing root is indicated by the Cauchy number *Ch* [34], defined as the ratio of the fluid dynamic pressure force to the structure elastic force, which can be expressed as follows:

(8) $$Ch=\frac{\rho_{air}A_{\phi }{}^{2}f^{2}{\bar{c}}^{3}b^{2}}{G}$$

where $\bar{c}$ is the mean wing chord length, *b* is the wingspan. The Cauchy number selected in this paper is 0.27 based on our previous research. Both the lift generation and power consumption are optimized at this Cauchy number.

The implicit method used to couple the fluid flow field with the motion of solid wings is briefly introduced here. At each time step, the pressure and shear in the flow field are obtained by solving the Navier–Stokes equations. Euler angular accelerations of the wings are updated from the wing torsional spring model which includes the aerodynamic forces, elastic forces, gravitational forces, and inertia forces. Velocities on the wings are then updated and applied back to the Navier–Stokes solver as boundary conditions to obtain an updated flow field. This two-way coupling process for solving both the fluid dynamic and wing kinematics is iterated until the angular accelerations of the wings reach a convergence criterion.

### 2.4. Turbulence Generator

Odor plumes in nature are usually broken by turbulent eddies into intermittent filaments segmented by low- or zero-concentration blanks. The development of odor plumes under different turbulence intensity levels can be very different. To investigate the odor plume in turbulence, a turbulence generator was used to generate a discrete flow field whose power spectrum matches an arbitrary turbulent energy spectrum [35].

The turbulence energy spectrum used in the turbulence generator is the von Kármán–Pao spectrum [36], which is defined as:

(9) $$E\left(\kappa \right)=\alpha \frac{{u^{\prime }}^{2}}{\kappa_{e}}\frac{{\left(\kappa /\kappa_{e}\right)}^{4}}{{\left(1+\kappa /\kappa_{e}\right)}^{17/6}}\exp\left[-2{\left(\frac{\kappa }{\kappa_{\eta }}\right)}^{2}\right]$$

where *κ* is the wave number, $u^{\prime }$ is the velocity fluctuation, *κ_e_* is related to the wavenumber at which the energy is maximum and $\kappa_{\eta }=\varepsilon^{1/4}\nu^{-3/4}$ is the Kolmogorov wave number (smallest turbulence structures).

The synthetic velocity field represented using a Fourier series at point $\vec{x}$ is:

(10) $$\vec{u}\left(\vec{x}\right)=2\sum_{m=1}^{M}q_{m}^{}\cos\left({\vec{\kappa }}_{m}^{}{\vec{x}}^{}-\psi_{m}\right){\vec{\sigma }}_{m}$$

where *M* is the number of modes, *q_m_* is the amplitude, ${\vec{\kappa }}_{m}=\left(\kappa_{x,m},\kappa_{y,m},\kappa_{z,m}\right)$ is the unit vector of the *m*^th^ wave number, *ψ_m_* is the phase angle, ${\vec{\sigma }}_{m}=\left(\sigma_{x,m},\sigma_{y,m},\sigma_{z,m}\right)$ is a unit direction vector.

**Figure 3:**
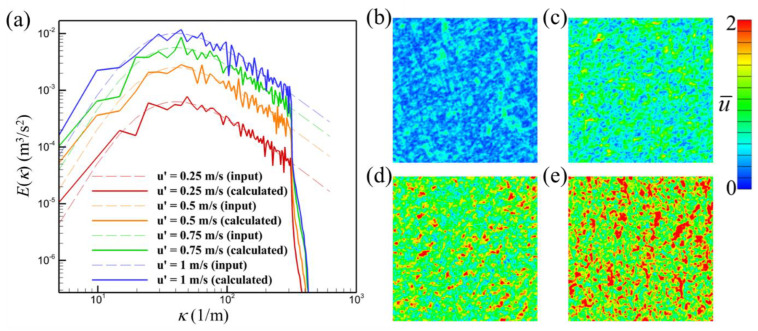
Spectra of synthetic turbulence field using the von Kármán–Pao spectrum as an input (dashed lines) with different velocity fluctuations (**a**), contour of velocity magnitude of the synthetic turbulence field with velocity fluctuation of 0.25 m/s (**b**), 0.5 m/s (**c**), 0.75 m/s (**d**), and 1 m/s (**e**). The grid resolution is 128 × 128 × 128 with a grid size of 0.01 m.

The turbulence generator calculates the coefficients in Equation (10) and reproduces the velocity field that matches the predefined turbulence energy spectrum. Figure 3 shows an example turbulence field generated by the turbulence generator.

The turbulence generator was used to generate the desired synthetic velocity field that contains small eddies that distort the odor plume structure to mimic odor plumes in nature. The velocity field was then added to the inlet of the computational domain. The development of the velocity field and odor landscape was solved using the Navier–Stokes equation solver and odor advection–diffusion equation solver.

### 2.5. Simulation Setup

To study how turbulent flow alters the olfactory performance of fruit flies during odor tracking flight, the current simulation adopts a fruit fly model that was put into a numerical wind tunnel to mimic a forward flight with constant incoming velocity. The odorant transport phenomenon is characterized by two non-dimensional parameters, Reynolds number (Re) and Schmidt number (Sc). The Reynolds number Re is 180 defined as Re=U¯tipb/ν, where ${\bar{U}}_{tip}$ = 3.2 m/s is the average wingtip velocity calculated from real fruit fly data [37]. The current setup results in a Reynolds number of 180. The Schmidt number Sc, defined as Sc=ν/D, the ratio of kinematic diffusivity of molecular diffusivity, ranging from 0.5 to 100, is the indicator of odorant diffusion intensity. This Schmidt number range covers most odorant particles in air, including NH_3_ (0.57), H_2_O (0.66), O_2_ (0.84), CH_4_ (0.99), CO_2_ (1.14), methanol (1.14), ethanol (1.5), and n-octane (3.2). These Schmidt numbers are smaller than most odorant particles in water, including O_2_ (340), NH_3_ (360), CO_2_ (410), ethanol (540), and CH_4_ (570). A smaller value of Sc means molecular diffusion dominates the odor transport, while a larger value of Sc means momentum diffusion dominates the odor transport. The advance ratio $J=U_{\infty }/U_{tip}$ in this study is 0.315, where the incoming air velocity *U_∞_* is 1.01 m/s, and the mean wing tip velocity *U_tip_* is 3.2 m/s.

To understand the effects of the two major parameters that dominate the odor transport phenomenon in turbulent flow, turbulent intensity *Tu* and Schmidt number Sc, simulations of odor transport were conducted in this study. As the indicator of the intensity of the turbulence fluctuation, the turbulence intensity *Tu*, defined as $Tu=u^{\prime }/U_{\infty }$ ranges from 0 to 0.9. Other parameters are kept identical in the following sections, including the advance ratio and flapping frequency.

**Figure 4:**
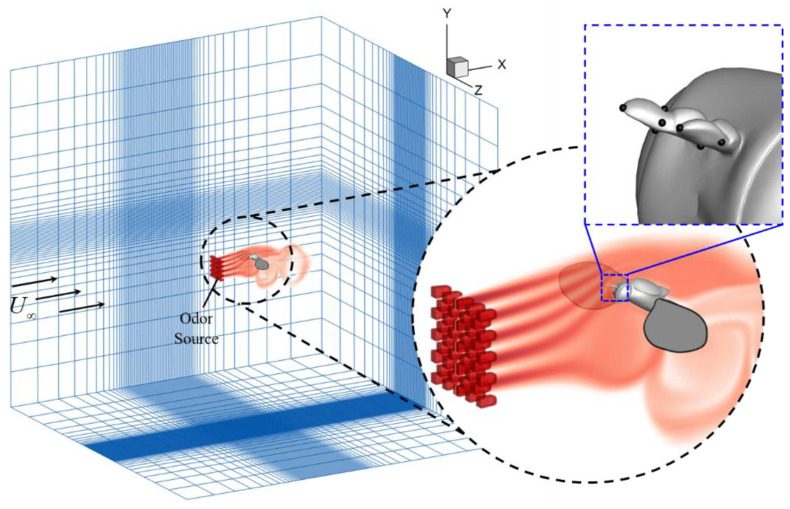
Case setup for simulating fruit fly odor tracking flight. The simulation was performed in a 320 × 128 × 240 (9.8 million) cell nonuniform Cartesian grid. An odor source with staggered arrangement was placed in front of the fruit fly model to simulate odor plumes. At the odor source the normalized odor concentration C* = 1. On each of the two antennae of the fruit fly, the average value of five sampling points was used to represent the odor detectability.

As shown in Figure 4, the simulations were performed in a 9.8-million-cell nonuniform Cartesian grid. An odor source with a staggered arrangement was placed in front of the fruit fly model. In the following section, the odor sensitivity is presented in terms of normalized odor concentration C* defined as

(11) $$C^{*}=C/C_{H}$$

where *C_H_* is the odor concentration at the odor source. At the odor source, the normalized odor concentration C* = 1.

At the odor source, a pre-generated turbulence field using the turbulence generator described in Section 2.4 was applied to perturb the odor plumes. On each of the two antennae of the fruit fly, the average value of C* of the five virtual sampling points was used to represent the odor sensitivity of the antenna. The computational domain size of the simulation is $30\bar{c}\times 30\bar{c}\times 30\bar{c}$ in terms of mean wing chord length ($\bar{c}$). The grid resolution is 320 × 128 × 240. The domain has several levels of refinement. The finest resolution is specified in a cuboidal region immediately around the insect. Outside of this region, there is a secondary fine resolution layer. Beyond the secondary layer, there is a coarse stretched layer. Boundary conditions on all walls of the computational domain were set as Neumann boundary conditions, except the Dirichlet boundary condition, which was set on the inlet wall with constant incoming velocity and odor intensity.

### 2.6. Evaluations of Olfactory Performance

**Figure 5:**
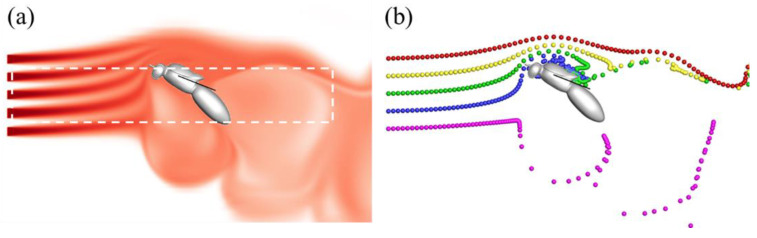
(**a**) Odor landscape computed by solving the advection–diffusion equation for a passive scalar. The domain of the odor concentration field marked by the white dashed rectangle is used to calculate the normalized odor concentration average value and its standard deviation and (**b**) streaklines released at the odor source are calculated using a Lagrangian approach.

To characterize the intensity of the odor plume and its fluctuation perturbed by the turbulence flow field and wing-induced flow, we calculated the mean value and standard deviation of normalized odor concentration C* inside the domain shown in Figure 5a along the flow direction. The mean value of odor concentration indicates the bulk odor intensity. The standard deviation denotes the fluctuations of odor concentration in space. Due to diffusion and mixing effect of turbulence, both values are expected to decrease along the flow direction.

Turbulent flow and the wingbeat-induced flow significantly perturb the odor landscape. Odor plumes are distorted and mixed by turbulent eddies. To visualize the development of odor plumes, streaklines were calculated and plotted to track the convective transport of odorant particles released at the odor source, as shown in Figure 5b. Streaklines are particles that are continuously released at the upstream flow. Trajectories of these particles were calculated using a Lagrangian approach. The velocities and locations of these particles were interpolated and calculated using a fourth-order Runge–Kutta method. Significant similarities between the odor landscape (Figure 5a) and streaklines (Figure 5b) were observed since they both represent the odor plume structure. Differences are present, however, due to the governing equations for each tracer. Calculating the odor landscape requires solving both advection due to fluid velocity and diffusion due to odor concentration gradients. Streaklines, on the other hand, only depend on the velocity field and are independent of diffusion. The fact that the two odor field markers shown in Figure 5 are similar is indicative of the fact that the turbulent velocity transport is dominant compared to molecular diffusion in this case.

## 3. Results and Discussion

In this section, we present numerical simulations that explore the structure of the odor landscape, influenced by both pre-generated turbulence fields and wingbeat-induced flows. These simulations employ the turbulence generator outlined in Section 2.4. Specifically, we focus on investigating the impact of two key parameters: turbulence intensity (Tu) and Schmidt number (Sc). In Section 3.1, we examine the effects of varying *Tu* levels, ranging from 0 to 0.9, while keeping Sc constant at 10. Conversely, in Section 3.2, we explore the influence of Sc values that span from 0.5 to 100, conducting simulations under two turbulence intensities: *Tu* = 0 and *Tu* = 0.3. To ensure the simulations reach a periodic state, each is carried out over a duration of seven flapping cycles.

### 3.1. Effects of Turbulence Intensity

**Figure 6:**
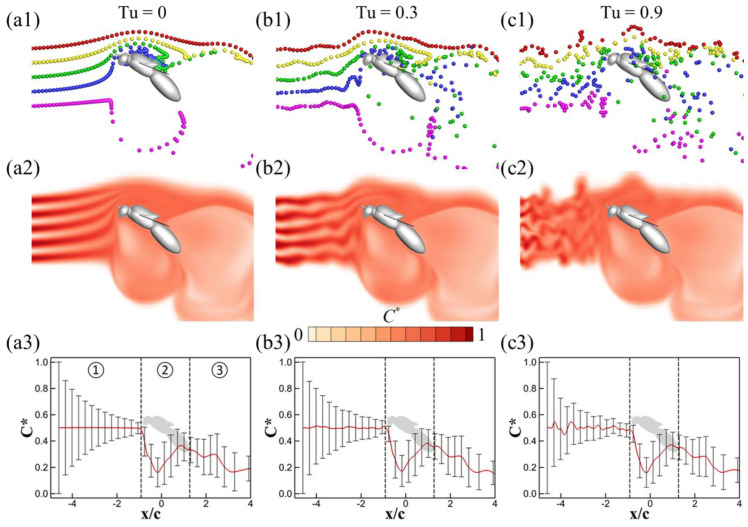
Visualizations of the odor landscape under varying conditions of turbulence intensity (*Tu*) at the mid-downstroke phase. Streaklines originating from the odor source and odor concentration contours are displayed on the symmetry plane. Panels (**a1**,**a2**,**a3**) correspond to *Tu* = 0; (**b1,b2**,**b3**) to *Tu* = 0.3; and (**c1**,**c2**,**c3**) to *Tu* = 0.9. Mean normalized odor concentrations and standard deviations along the x-direction are presented in panels (**a3**,**b3**,**c3**). The domain for these calculations is delineated in Figure 5. Vertical dashed lines in panels (**a3**,**b3**,**c3**) mark the positions of the antennae and tail, with x/c = 0 representing the wing root.

To elucidate the effects of turbulence on the odor landscape, Figure 6 presents side-view visualizations of streaklines and odor concentration contours on the symmetry plane at mid-downstroke for different turbulence intensities: *Tu* = 0, 0.3, and 0.9. The Schmidt number in these scenarios is fixed at Sc = 10. The turbulence significantly disrupts the odor plumes. In the absence of turbulence (*Tu* = 0), both the streaklines and the odor landscape exhibit a well-organized pattern. Odor plumes are emitted from the source flow in alignment with the incoming air stream and are subsequently deflected by the wingbeat-induced flow. At *Tu* = 0.3, the turbulence introduces perturbations to the streaklines and the odor landscape; however, these perturbations are relatively modest due to the low level of turbulence compared to the incoming airflow velocity. At *Tu* = 0.9, turbulence substantially disrupts both the streaklines and the odor landscape, as shown in Figure 6(c1,c2). The streaklines and odor plumes appear distorted and substantially mixed by turbulent eddies. Notably, when *Tu* > 0, as seen in Figure 6(b1,c1), the streaklines become corrugated and deviate from the symmetry plane, unlike the well-aligned streaklines in the Tu = 0 scenario (Figure 6(a1)).

Figure 6(a3,b3,c3) displays the mean values and standard deviations of odor concentration for *Tu* = 0, 0.3, and 0.9 at mid-downstroke. For ease of comparison, the flow field is segmented into three distinct regions: the upwind region (①), the wing-induced flow region (②), and the downwind region (③). The general trends in odor concentration along the flow direction appear consistent across all values of *Tu*. Specifically, in the upwind region (①), an increase in *Tu* results in noticeable fluctuations in the mean normalized odor concentration, although the standard deviations remain relatively stable across varying levels of *Tu*. Intriguingly, while there are discernible differences in the upwind region (①), the variations are almost negligible in both the wingbeat-induced flow region (②) and the downwind region (③). This could be attributed to the fact that the influence of turbulence is relatively minor compared to the wingbeat-induced flow (also see Table 1). As previously reported by Lei and Li [38], wingbeat-induced flows actively trap odor plumes over a broad area towards the antennae, thereby diminishing the impact of turbulence-induced perturbations on the odor plumes. Notably, all three cases exhibit a sudden drop in odor concentration within the wing-induced flow region (②). This corresponds to a large area of low odor concentration observed in the odor landscapes in Figure 6(a2,b2,c2), situated just anterior to the insect’s thorax. This phenomenon occurs because the flapping wings draw in air with a low odor concentration from the surroundings. Our observations also demonstrate that the antennae of insects are well positioned to receive odor stimuli while avoiding significant air disturbance compared to other locations along the body.

**Figure 7:**
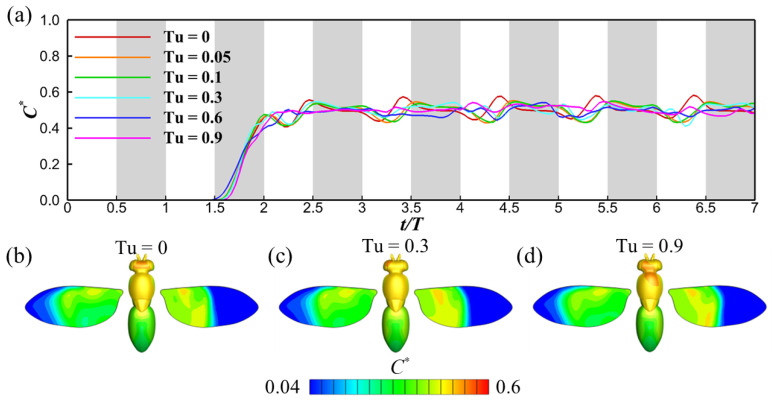
Time history of the normalized odor concentration C* for different turbulence intensities (**a**), odor concentration contour on the fruit fly for *Tu* = 0 (**b**), 0.3 (**c**), and 0.9 (**d**). The Schmidt number for these cases is Sc = 10. The grey shaded regions denote downstrokes. For the odor concentration distribution on the fruit fly (**b**–**d**), the left wing shows the odor concentration on the upper surface of the wing, the right wing shows the odor concentration on the lower surface.

Since the wingbeat-induced flow exerts a much stronger influence than turbulence fluctuations, the impact of varying turbulence intensities is nearly inconsequential in both the wing-induced flow region (②) and the downwind region (③). Our primary focus, however, lies in understanding the odor sensitivity at the antennae, which serves as a transitional zone between the upwind region (①) and the wing-induced flow region (②). Figure 7a illustrates the temporal evolution of normalized odor sensitivity (C*) at the antennae across different turbulence intensities (*Tu*). Intriguingly, as *Tu* increases, a decrease in the amplitude of C* fluctuations is observed. This is likely due to the turbulence-induced dispersion and mixing of the odor plumes, which make the concentration of the odorant more spatially uniform in the region directly upstream of the antennae. In the absence of turbulent mixing, the odorant is organized into regions of high and low concentration, as seen in Figure 6(a2). When the flow is drawn over the antennae by the wing beats, this coherent structure is retained and manifests in the peaks and troughs in the time trace in Figure 7a. When *Tu* is higher, the coherence is destroyed by turbulent mixing, and so these fluctuations are no longer observed. It is worth noting, however, that other spatial fluctuations in odor distribution manifest at higher *Tu* levels. Figure 7b–d depict the odor concentration contours on the insect’s body and wings. Across all cases, these contours remain remarkably similar with one notable exception—the antennae region. Here, the contours display noticeable asymmetry (Figure 7c,d). A video offering a close-up view that showcases the asymmetrical feature of odor perception is available in the Supplementary Materials. We posit that the asymmetry is due to the interaction between the wing-induced flow and turbulent velocity field. While the turbulence enhances mixing and therefore creates a more spatially uniform odor plume upstream of the insect, providing a more consistent temporal signal to the antennae, turbulent velocity fluctuations couple with the wing-induced flow to create spatial variations in the distribution of the plume around the antennae. Therefore, the antennae emerge as a unique locus on the insect’s body capable of detecting subtle shifts in odor concentration attributable to turbulence fluctuations.

The interaction between odor plumes, turbulence fluctuations, and wingbeat-induced flow is a complex yet crucial aspect of insect olfaction. Our results indicate that turbulence significantly distorts and mixes odor plumes in the upwind region (①), echoing findings from previous studies that have examined the role of environmental turbulence in chemical plume dispersion [7,11]. These perturbed plumes are then actively drawn toward the antennae by strong wingbeat-induced flows. Consequently, the antennae can sense these spatial fluctuations, thereby providing the insect with vital olfactory cues for navigation. In contrast, the influence of turbulence intensity appears to be nearly inconsequential in the wing-induced flow region (②) and downwind region (③). This observation is consistent with studies suggesting that strong local flows, such as those generated by wing flapping, can overshadow environmental turbulence effects [29]. Flapping-wing insects typically exhibit advance ratios ranging between 0.3 and 0.5, as shown in Table 1. This implies that their mean wing-tip velocity is at least twice their average flight speed. Given these kinematics, wingbeat-induced flow significantly impacts both the flow field and odor distribution around the insect’s body, including its olfactory receptors—the antennae. In a natural flight setting (assuming the turbulence intensity is smaller than their flying speed), our simulations suggest that, compared to background turbulence, wingbeat-induced flow disturbances are more likely to play a pivotal role in insect olfactory perception. While our study provides the insights, further biological investigations are essential to validate and expand upon these findings. Future work could also examine how these fluid dynamic interactions translate into behavioral responses in real-world scenarios, contributing to a more comprehensive understanding of insect navigation and sensory biology.

### 3.2. Effects of Schmidt Number

The Schmidt number is a critical factor influencing the development of odor plumes. In this part of our study, we investigate its effects over a range from 0.5 to 100. Initially, to isolate the role of the Schmidt number, we examine the odor concentration field under conditions of zero turbulence intensity (*Tu* = 0). Following this, we extend our analysis to include a moderate level of turbulence (*Tu* = 0.3) to assess how turbulence fluctuations interact with variations in the Schmidt number.

**Figure 8:**
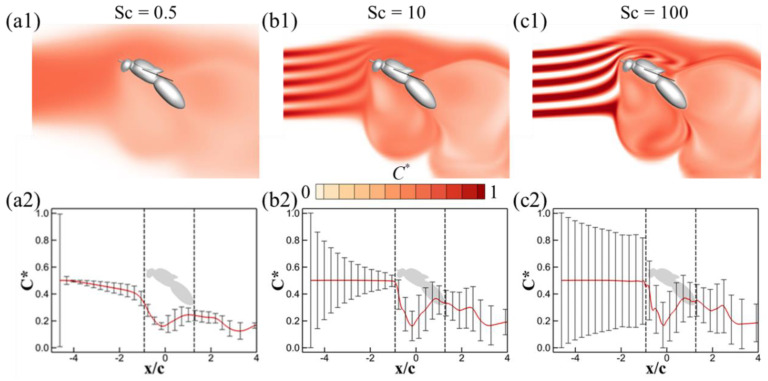
Odor concentration contour on the symmetry plane, mean normalized odor concentration and the standard deviation along the *x*-direction for Sc = 0.5 (**a1**,**a2**), Sc = 10 (**b1**,**b2**), Sc = 100 (**c1**,**c2**) at mid-downstroke. For the average odor concentration and the standard deviation, the calculation domain is shown in Figure 5. The turbulence intensity for these cases is *Tu* = 0. The vertical dashed lines in (**a2**,**b2**,**c2**) denote the locations of antennae and tail. *x/c* = 0 is the wing root.

Figure 8 illustrates the odor concentration contours on the symmetry plane, as well as the corresponding mean and standard deviation of odor concentrations at mid-downstroke for Schmidt numbers Sc = 0.5, 10, and 100, in a turbulence-free environment (*Tu* = 0). Given that the Schmidt number does not affect the flow field, the streaklines for all these scenarios at *Tu* = 0 align with those depicted in Figure 6(a1). For the low Schmidt number scenario (Figure 8(a1), Sc = 0.5), the rapid dispersion of odorant molecules predominates, leading to a swift dilution of odor plumes into the surrounding air. As evident from Figure 8(a2), the mean normalized odor concentration in the upwind region declines along the flow direction, while the standard deviation remains notably compact. Conversely, at a high Schmidt number (Sc = 100), advection becomes the dominant mechanism driving odor transport. Figure 8(c2) shows a slower reduction in the standard deviation of normalized odor concentration (C*) in the upwind region when compared to the scenario with Sc = 10. Despite these variations in Schmidt number, the profiles of odor concentration in the wingbeat-induced flow region ② and downwind region ③ remain strikingly similar across all cases. This similarity underscores the overpowering influence of the wingbeat-induced flow, which appears to mitigate the variances introduced by differing Schmidt numbers.

**Figure 9:**
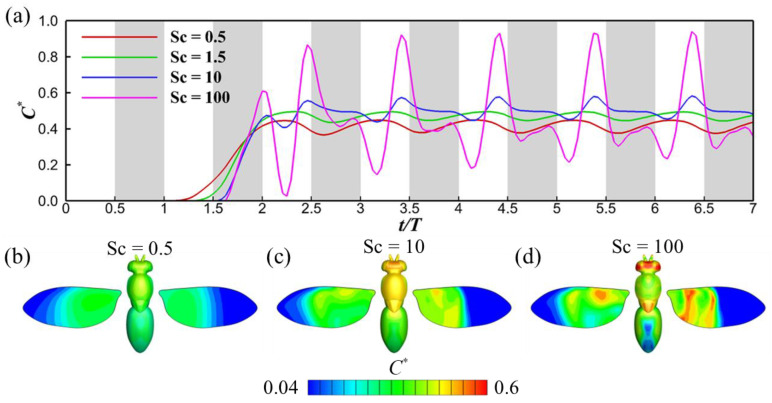
Time history of the normalized odor concentration C* for different Schmidt numbers (**a**); odor concentration contour on the fruit fly for Sc = 0.5 (**b**), 10 (**c**), and 100 (**d**). The turbulence intensity for these cases is *Tu* = 0. The grey shaded regions denote downstrokes. For the odor concentration distribution on the fruit fly (**b**–**d**), the left wing shows the odor concentration on the upper surface of the wing, and the right wing shows the odor concentration on the lower surface.

Figure 9a presents the odor concentration experienced at the antennae for scenarios with no turbulence (*Tu* = 0). A pattern of periodic fluctuations in odor concentration is apparent, stemming from the periodic perturbations introduced by wing-induced flow. Notably, for Sc = 0.5, the odor sensitivity is substantially lower compared to other Sc values, attributable to its greater diffusivity. As the Schmidt number increases, the rate of odor dissipation diminishes, leading to advection becoming the primary driver of odor transport. Consequently, the extent of odor sensitivity fluctuations (induced by wingbeat-generated flows) escalates. This is analogous to the situation analyzed in Section 3.1, except that, in this case, enhanced uniformity in the odor plume upstream of the insect is due to increased scalar diffusion, instead of turbulent mixing. For a high Schmidt number, the distribution of the odorant retains its original spatial distribution far downstream of the odor source, resulting in significant temporal fluctuations in the signal detected by the antennae. This pattern suggests that insects could potentially distinguish between different odorant volatiles within a mixed emission from the same odor source, simply by comparing fluctuations in odor concentration. Again, this hypothesis may need more experimental evidence to be proven.

Turning to Figure 9b–d, these depict the odor concentration contours on the insect’s body surface. The contours parallel the observed trends in both the odor landscape and odor detectability. For smaller Schmidt numbers, as illustrated in Figure 9b, the extensive odor diffusivity renders the odor landscape nearly homogeneous, resulting in smoothly varying odor concentration contours. Conversely, at higher Schmidt numbers, such as in Figure 9d, the contours display fluctuations, and the overall odor concentration on the insect’s surface is markedly elevated. A close-up video highlighting the instantaneous odor concentration contours is available in the Supplementary Materials.

**Figure 10:**
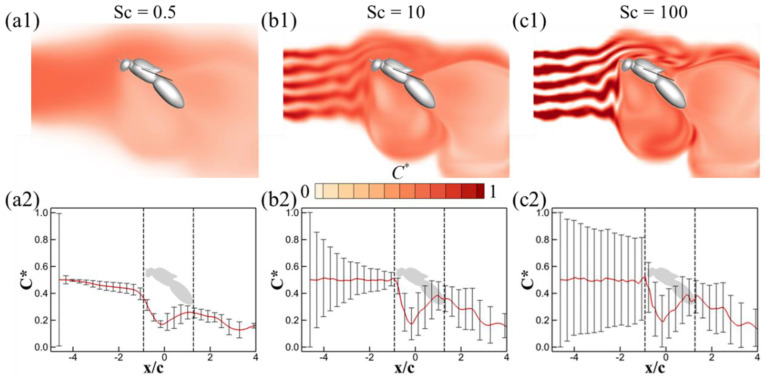
Odor concentration contour on the symmetry plane, mean normalized odor concentration and the standard deviation along the *x*-direction for Sc = 0.5 (**a1**,**a2**), Sc = 10 (**b1**,**b2**) and Sc = 100 (**c1**,**c2**) at mid-downstroke. For the average odor concentration and the standard deviation, the calculation domain is shown in Figure 5. The turbulence intensity for these cases is *Tu* = 0.3.

Figure 10 provides a visualization of the odor concentration contours on the symmetry plane, along with the corresponding mean values and standard deviations of odor concentration for Sc = 0.5, 10, and 100 during mid-downstroke, with turbulence set at *Tu* = 0.3. The associated streaklines for these scenarios can be referenced in Figure 6(a2). In the case of Sc = 0.5, the dominance of odor diffusion in shaping the odor landscape means that turbulence fluctuations have a negligible impact, echoing observations in the *Tu* = 0 scenario as depicted in Figure 8(a1,a2). Conversely, for Sc = 10 and 100, visible distortions in the structure of the odor plumes are apparent (as shown in Figure 10(b1,c1)), along with noticeable fluctuations in the mean normalized odor concentration in the upwind region ① (Figure 10(b2,c2)). Despite these variations in the upwind region, the similarities are far more pronounced in the wingbeat-induced flow region ② and the downwind region ③. Here, the strong wingbeat-induced flow actively channels the odor plumes, rendering the turbulence-induced differences less significant.

**Figure 11:**
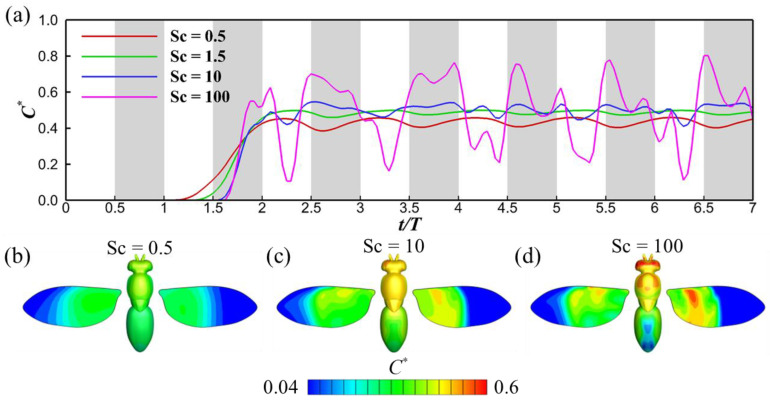
Time history of the normalized odor concentration C* for different Schmidt numbers (**a**); odor concentration contour on the fruit fly for Sc = 0.5 (**b**), 10 (**c**), and 100 (**d**). The turbulence intensity for these cases is *Tu* = 0.3. The grey shaded regions denote downstrokes. For the odor concentration distribution on the fruit fly (**b**–**d**), the left wing shows the odor concentration on the upper surface of the wing, and the right wing shows the odor concentration on the lower surface.

To delve deeper into how turbulence fluctuations influence odor concentration at the antennae, we illustrated the temporal progression of normalized odor concentration (C*) for various Schmidt numbers at *Tu* = 0.3, as shown in Figure 11. In contrast to the *Tu* = 0 scenarios depicted in Figure 9, striking disparities in odor concentration are evident at higher Schmidt numbers (Sc = 10 and 100). Due to the turbulence-induced perturbations in the structure of the odor plumes, the odor concentration fails to stabilize into a repeating pattern, unlike what is observed in the *Tu* = 0 cases because the turbulent fluctuations in the flow and the original spatial distribution of the odor source are on comparable scales. We note that the magnitude of the fluctuations in the Sc = 10 case are significantly less in the situation with *Tu* = 0.3 compared to *Tu* = 0, because the turbulent mixing serves to diffuse the odorant. Molecular diffusion alone is strong enough in the cases with lower Schmidt number that the turbulence has little effect in this regard. Additionally, a greater number of more minor fluctuations in odor sensitivity are discernible in the cases with higher Schmidt number due to the enhanced turbulent advection. Figure 11b–d present the odor concentration contours on the insect’s surface. The most salient difference when compared to the *Tu* = 0 cases is the loss of symmetry in the odor concentration contours across the insect’s body. A video in the Supplementary Materials provides a detailed view highlighting the asymmetrical nature of odor perception at *Tu* = 0.3.

The Schmidt number serves as a critical variable, altering odorant diffusivity and thereby affecting odor sensitivity. Pronounced fluctuations in odor concentration become evident at higher Schmidt numbers, suggesting that insects might capitalize on this phenomenon to distinguish between various odorant volatiles emanating from different odor sources. While turbulence fluctuations generally diminish the amplitude of concentration fluctuations, they can enhance their frequency depending on the frequency of the turbulent fluctuations themselves, especially at higher Schmidt numbers.

The above findings extend beyond the role of turbulence intensity, shedding light on the crucial impact of the Schmidt number (Sc) on olfactory perception. In our simulation, we explored a relatively broad range of Schmidt numbers (0.5–100) to delineate how odor distribution patterns are affected by flow disturbances. At lower Schmidt numbers (e.g., Sc = 0.5), turbulence intensity exerts minimal influence on the odor landscape because the molecular diffusion essentially overshadows the mixing effect of turbulence. Conversely, at higher Schmidt numbers (Sc = 100), our simulations reveal marked alterations in the odor distribution field. Given that the Schmidt numbers in air are relatively low, while in aquatic environments they can surge to several hundred, the transport phenomena of odorants at differing Schmidt numbers suggest that terrestrial and aquatic animals may rely on distinct olfactory search mechanisms to navigate their respective environments. However, it is interesting to note that their observed odor tracking behaviors often appear remarkably similar [2].

Another insight gleaned from our simulations pertains to the variations in mass diffusivity (D) for different odors within the same fluid medium. For example, an odor that is particularly attractive to certain insects may have a mass diffusivity (D) value around a specific parameter. In contrast, another odor, which appeals to a different set of animals, may exhibit a considerably different D value. These disparate D values contribute to varying Schmidt numbers—leading to unique odorant transport phenomena within the fluid medium. Consequently, the odor-tracking behaviors exhibited by different species may be partially attributable to these divergent transport mechanisms. While this hypothesis offers a compelling avenue for exploration, empirical validation is essential to corroborate these theoretical insights. We suggest that experiments could be performed to help further elucidate the effects of mass diffusivity and turbulence intensity, in which the mass diffusivity of an odorant is varied, e.g., by dispersing the odorant in liquid micro- or nano-droplets vs. directly into the air. The turbulence intensity can be varied by using grid generators with different spacings. PIV measurements would add significant value to such experiments, by providing experimental evidence to support our conclusions about the role of wing-induced flow in olfactory sensing and its effect dominating over free stream turbulence.

## 4. Conclusions

In this study, we employed a three-way coupled immersed-boundary-method-based CFD solver to examine the olfactory experience of a fruit fly engaged in an upwind surging odor-tracking flight. The Navier–Stokes equations are strongly coupled with equations of motion to simulate the passive flapping wings, along with the surrounding airflow. Concurrently, advection–diffusion equations are solved at each time step to derive the evolving odor concentration field.

Our research offers a comprehensive computational lens into the intricate relationship between turbulence intensity and Schmidt number in shaping the olfactory landscape for flapping-wing insects. Our simulations reveal that turbulence fluctuations have a pronounced impact on the fragmentation and dispersion of odor plumes in the upwind region. These fragmented plumes are subsequently channeled by the potent wingbeat-induced flow towards the insect’s antennae, which are sensitive to these odor concentration fluctuations. Our findings also highlight the critical role that the Schmidt number (Sc) plays in the transport and diffusion dynamics of odorants. At lower Sc values, diffusion is the dominant mechanism, leading to a rapid dispersion of the odor plume. In contrast, higher Sc values result in advection-dominated transport, thereby preserving the structural integrity of the plume. This divergence in transport phenomena across different Sc ranges suggests that distinct olfactory search strategies may be required for animals in aerial versus aquatic environments. Additionally, our study implies that the specific mass diffusivities of different odors result in unique Schmidt numbers, offering a potential explanation for the observed variability in odor-tracking behaviors across animal species. Overall, our findings pave the way for future empirical studies that aim to better understand how complex fluid dynamics interact with biological olfactory systems across various media—whether air or water. They also hold promise for the development of odor-guided robotic systems that emulate biological olfaction mechanisms.

## Figures and Tables

**Table 1:** Comparison of flying speeds, mean wingtip velocities, advance ratios, and Reynolds numbers for forward-flying insects known for their proficiency in odor source tracking for survival.

Insects	Forward Flying Speed ($U_{\infty }$)	Wing Tip Velocity ($U_{tip}$)	Advance Ratio ($J=U_{\infty }/U_{tip}$)	Reynolds Number (*Re*)
Fruit fly (current)	1.01 m/s	3.2 m/s	0.315	180
Bumblebee [29]	2.5 m/s	8.75 m/s	0.286	2042
Butterfly [39]	1.14 m/s	2.79 m/s	0.41	3455
Hawkmoth [40]	2.0 m/s	4.88 m/s	0.41	7335

## Data Availability

The data that support the findings of this study are available from the corresponding author upon reasonable request.

## Supplementary Materials

The following supporting information can be downloaded at: https://www.mdpi.com/article/10.3390/biomimetics8080593/s1, See submitted supplementary video online.
